# IgA nephropathy: a review of existing and emerging therapies

**DOI:** 10.3389/fneph.2023.1175088

**Published:** 2023-05-23

**Authors:** Sahibzadi Mahrukh Noor, Farah Abuazzam, Roy Mathew, Zhiwei Zhang, Amir Abdipour, Sayna Norouzi

**Affiliations:** Department of Nephrology, Loma Linda University, Loma Linda, CA, United States

**Keywords:** IgA nephropathy, proteinuria, SGLT2 inhibitors, glucocorticoids, budesonide, sparsentan

## Abstract

IgA nephropathy (IgAN) is the most common form of primary glomerulonephritis worldwide. Recently, there have been multiple advances in the understanding of IgAN pathophysiology and therapeutic options. Despite the advent of new treatment options, individual risk stratification of the disease course and choosing the best treatment strategy for the patient remains challenging. A multitude of clinical trials is ongoing, opening multiple opportunities for enrollment. In this brief review we discuss the current approach to the management of IgAN and highlight the ongoing clinical trials.

## Introduction

IgA nephropathy (IgAN) was first described in 1968 by Berger and Hinglais (1). It is the most common primary glomerular disease (2) and a leading cause of chronic kidney disease (CKD) and end-stage renal disease (ESRD) worldwide (3–5). While being the most prevalent glomerulopathy, it is marked by remarkable geographic and ethnic heterogeneity, with incidence varying from 39–45 cases per million population (p.m.p.) per year in Japan to 10–21 cases p.m.p. per year in the USA, and with 9.9 cases p.m.p. per year in the UK (6). It accounts for approximately 40% of all native biopsies in Japan, 25% in Europe, 12% in the USA, and less than 5% in Central Africa (3). This might be attributed to obscure genetic and environmental factors, along with intensive urine screening and a low threshold for renal biopsies in Asian countries (4).

IgAN treatment has evolved at a rapid rate in the last decade. A number of recently completed and ongoing clinical trials are leading to a shift in the treatment paradigm. We aim to review the traditional treatment strategies, Kidney Disease: Improving Global Outcomes (KDIGO) 2021 guidelines, novel therapies, and ongoing clinical trials in this ever-changing field.

## Current treatment strategies

### Supportive care

As per the KDIGO 2021 guidelines, supportive care (SC) for IgAN involves rigorous blood pressure control, optimal renin–angiotensin–aldosterone system inhibition (RAAS-i), assessing and addressing cardiovascular risk, and lifestyle changes, including dietary sodium restriction, smoking cessation, weight control, and exercise, as appropriate (5). Other than dietary sodium restriction, no other specific dietary intervention has been shown to affect outcomes in IgAN.

Hypertension and proteinuria are independent risk factors for disease progression in IgAN. In a prospective study of 332 patients with IgAN, the cumulative incidence of dialysis or death was much higher in patients with hypertension at disease diagnosis (defined in the study as > 140/90 mmHg) than in those without hypertension (15% vs. 3% and 41% vs. 6% at 10 and 20 years, respectively) (7). In another study involving 542 patients with IgAN, a higher mean arterial pressure was associated with a higher risk of progressive kidney disease. This effect was observed at all levels of proteinuria (8). There are no specific blood pressure targets to improve progression; however, KDIGO recommends a blood pressure target of < 120/80 mmHg for all glomerular diseases.

In a study involving 1,155 patients, there was a statistically significant improvement in 10-year kidney survival in patients with proteinuria < 1 gm/day compared with patients with proteinuria >1 gm/day, with 10-year dialysis-free survival of 94% (95% CI 90% to 98%) and 20-year dialysis-free survival of 89% (95% CI 82% to 96%). Patients with proteinuria > 1.0 gm/day were associated with a 9.4-fold risk compared with patients with proteinuria < 1.0 gm/day (*p* < 0.001) and a 46.5-fold risk compared with patients with proteinuria < 0.5 gm/day (*p* < 0.001). Moreover, patients who achieved proteinuria < 0.5 gm/day benefit much more than those with proteinuria between 0.5 and 1.0 gm/day [hazard ratio (HR) 13.1, *p* < 0.001] (9). Similar results were seen in an individual participant-level meta-analysis of data for 830 patients from 11 randomized controlled trials (RCTs), which showed that a reduction in proteinuria (independent of the presence or absence of hypertension) was associated with a lower risk for doubling of serum creatinine level, ESRD, or death in patients with IgAN (10).

Retrospective data from large registries have revealed that patients with IgAN treated with an angiotensin-converting enzyme inhibitor (ACEi) to control blood pressure have a lower rate of annual loss of kidney function than similar patients not treated with an ACEi or an angiotensin II receptor blocker (ARB) (10). An RCT of 109 Asian patients with IgAN showed greater proteinuria reduction and slowing of the rate of kidney deterioration with valsartan compared with placebo (11). An RCT of 44 patients with IgAN comparing enalapril with other antihypertensives (nifedipine, amlodipine, atenolol, diuretics, and doxazosin) demonstrated better kidney survival and a reduction in proteinuria (12). However, there is a lack of studies evaluating the efficacy of renin-angiotensin-aldosterone system (RAAS) blockade in IgAN with moderately increased albuminuria (30–300 mg/d) and normal blood pressure.

Therefore, KDIGO guidelines recommend that all patients with proteinuria > 0.5 gm/day, irrespective of whether or not they have hypertension, be treated with either an ACEi or an ARB as a category 1B recommendation (1: we recommend, B: moderate evidence meaning the true effect is likely to be close to the estimate of the effect, but there is a possibility that it is substantially different). Maximally tolerated doses of RAAS inhibitors should be used. No benefit of dual blockade has been demonstrated (13). The treatment goal is proteinuria reduction to < 1 gm/day.

### Sodium-glucose cotransporter-2 inhibitors

Sodium–glucose cotransporter-2 inhibitors (SGLT2 inhibitors) have shown promising results in patients with CKD, including the DIAMOND, DAPA-CKD, and EMPA-KIDNEY trials (14). A prespecified analysis of the DAPA-CKD trial in 270 patients with IgAN (with 254 biopsy-confirmed cases) showed that adding SGLT2 inhibitors to angiotensin-converting enzyme (ACE)/ARB therapy substantially reduced the risk of CKD progression. The primary outcome [composite of sustained decline in estimated glomerular filtration rate (eGFR) of > 50%, end-stage kidney disease, or death from a kidney disease-related or cardiovascular cause) occurred in 4% of participants on dapagliflozin and 15% of participants on placebo (HR 0.29; 95% CI 0.12 to 0.73). Mean rates of eGFR decline with dapagliflozin and placebo were –3.5 and –4.7 mL/min/1.73 m^2^/year, respectively. Dapagliflozin reduced the urinary albumin-to-creatinine ratio by 26% relative to placebo. Adverse events leading to study drug discontinuation were similar with dapagliflozin and placebo (15). The EMPA-KIDNEY trial had 1,669 patients with glomerular disease, and thus a stratified analysis based on the etiology of glomerular disease will be highly anticipated.

KDIGO 2021 guidelines do not recommend SGLT2 inhibitors because the guidelines predate the above trials. With the above results, SGLT2 inhibitors are likely to become the standard of care in IgAN worldwide; however, with the caveat that the trials (previous and ongoing) do not universally include combination of RAAS-i and sodium-glucose cotransporter-2 (SGLT2) inhibition.

### Sparsentan

Sparsentan is an oral dual endothelin and angiotensin II receptor antagonist that was recently approved by the Food and Drug Administration (FDA) for high-risk IgAN patients. The Phase III PROTECT trial compared sparsentan 400 mg total with irbesartan 300 mg total in 404 patients with proteinuria ≥ 1 gm/day after a 3-month run-in period (16). The urine protein-to-creatinine ratio (UPCR) decreased by 49.8% (mean percentage decrease from baseline) in the sparsentan group compared with 15.1% in the irbesartan group at 36 weeks (between-group relative reduction of 41%, *p* < 0·0001). Adverse events were similar in both groups, with no cases of hepatotoxicity, heart failure, or edema-related discontinuations. Liver aminotransferases and total bilirubin should be checked prior to initiation of treatment and alanine transaminase and aspartate transaminase should be monitored monthly for 12 months followed by every 3 months during treatment. Treatment should be held if aminotransferases increase to three times the upper limit of normal. Since animal data have shown major birth defects associated with sparsentan use in pregnancy, pregnancy testing is required before, during, and after treatment, and women of child-bearing age must use effective contraception prior to initiation of treatment, during treatment, and for 1 month after treatment.

### Hydroxychloroquine

Hydroxychloroquine is recommended only for Chinese patients who remain at high risk of disease progression despite optimized SC. An RCT in China showed a 48% reduction in proteinuria, compared with a 10% reduction with a placebo, after 6 months of use in patients with 0.75–3.5 gm/day proteinuria, despite optimized RAAS-inhibition (17).

## Immunosuppression

### Glucocorticoids

Patients with persistent proteinuria > 1 gm/day despite optimized SC (defined as treatment with a maximal tolerated or allowed daily dose of RAAS blockade and good blood pressure control) for a minimum of 3–6 months are at high risk of progressive CKD and need to be evaluated for further treatment options. The MEST-C Oxford classification (18) and International IgA Nephropathy Prediction Tool (IIgAN-PT) quantify the risk of progression to ESRD; however, there is no uniform threshold above which a patient is considered a candidate for immunosuppressive therapy.

Multiple RCTs have evaluated the effect of glucocorticoids in IgAN (**Table 1**) with varying results. During the earlier studies, RAAS blockade was not the standard of care, and therefore some of the trial results cannot be extrapolated to the current IgAN population where RAAS-i is universal.

**Table 1:** Clinical trials evaluating steroid use in IgAN.

Study	Inclusion criteria	Number of patients	Follow-up (months)	Steroids	Control	Results
Lai et al., 1986 (19)	IgAN patients with nephrotic syndrome	34	38	Prednisone 40–60 mg/day	No treatment	- Reduction in proteinuria - No effect on eGFR
Julian and Barker, 1993 (20)	- eGFR > 25 mL/min/1.73 m^2^ - Proteinuria > 1.0 gm/day - Chronic lesions in renal specimen	31	6–24	Prednisone 60 mg daily every other day	No treatment	- No change in proteinuria - Improved renal survival (i.e., lower creatinine)
Shoji et al., 2000 (21)	- Creatinine < 1.5 mg/dL - Proteinuria < 1.5 gm/day	19	12	Prednisone 0.8 mg/kg/day	Dipyridamole 300 mg daily	- Reduction in proteinuria - No effect on eGFR - Improvement in renal histologic lesions
Katafuchi et al., 2003 (22)	- Creatinine < 1.5 mg/dL	90	65	Prednisolone 20 mg/day	Dipyridamole 150–300 mg daily	- Reduction in proteinuria - No change in renal survival (i.e., ESRD)
Pozzi et al., 2004 (23)	- Creatinine < 1.5 mg/dL - Proteinuria 1–3.5 gm/day	86	82	Methylprednisolone 1 g for 3 days followed by prednisone 0.5 mg/kg every other day	Supportive	- Reduction in proteinuria - Improved renal survival (i.e., doubling of creatinine) - No serious adverse events
Hogg, 2006 (24)	- Proteinuria > 1.0 or > 0.5 with high-risk renal lesions - eGFR > 50 mL/min/1.73 m^2^	64	24	- Prednisone 60 mg every other day	Placebo	- No change in proteinuria - No change in renal survival (i.e., 60% reduction of eGFR)
Lv et al., 2009 (25)	- Proteinuria 1–5 gm/day - eGFR > 30 mL/min/1.73 m^2^	63	27.3	Prednisone 0.8–1 mg/kgm/day plus RAAS-i	Cilazapril 3.75 mg daily	- Reduction in proteinuria - Improved renal survival (i.e., doubling of creatinine)
Manno et al., 2009 (26)	- Proteinuria > 1 gm/day - eGFR > 50 mL/min/1.73 m^2^	97	96	Prednisone 1 mg/kgm/day plus RAAS-i	Ramipril 7.5 mg daily	- Reduction in proteinuria - Improved renal survival (i.e., doubling of creatinine and ESRD)

The two latest RCTs evaluating glucocorticoids in IgAN are the STOP-IgA and TESTING trials. The STOP-IgA trial had a 6-month run-in period followed by intravenous methylprednisolone 1 gm/day for 3 days at the start of months 1, 3, and 5, and oral prednisolone 0.5 mg/kg/48 h on the other days in patients with eGFR > 60 mL/min/1.73 m^2^; and used oral prednisone 40 mgm/day (tapered to 10 mgm/day over the first 3 months, 10 mgm/day during months 4–6, and 7.5 mgm/day during months 7–36), along with cyclophosphamide 1.5 mg/kgm/day for 3 months, followed by azathioprine 1.5 mg/kgm/day during months 4–36, in patients with eGFR 30–59 mL/min/1.73 m^2^. After a median follow-up of 7.4 years, steroids did not alter long-term outcomes; 45% of the steroid group reached the end point of 40% eGFR loss, ESRD, or death compared with 50% of the immunosuppression group (HR 1.2, 95% CI 0.75 to 1.92). ESRD developed in 23.6% of patients in the supportive group and 25.9% of patients in the immunosuppression group (27).

In the STOP-IgA trial, mean eGFR was 59 mL/min/1.73 m^2^and mean proteinuria was 1.7 gm/day at the start of the trial (2.2 gm/day at the initiation of run-in phase). The mean annual eGFR loss was 2.5 mL/min/1.73 m^2^/year compared with 5–6 mL/min/1.73 m^2^/year in previous RCTs, which could be attributed to intensive SC for 6 months prior to randomization and during the trial. The slow decline in eGFR also indicates that STOP-IgA patients were at low risk of progressive kidney disease. A retrospective analysis of MEST-C histologic grading in 47% of patients (histologic grading was available for 70/149 patients only) revealed that both the primary end point and ESRD were more likely in patients with M1, E1, S1, and T1/2 than in patients with zero scores in the respective MEST-C categories. However, none of the MEST-C categories was significantly associated with annual eGFR loss. According to the authors, due to statistical and analytical limitations, the study was unable to define/identify subgroups based on kidney biopsy histology grading, baseline eGFR, or proteinuria level, which might have benefited from immunosuppression (28).

The original TESTING trial compared placebo with oral methylprednisolone (0.6–0.8 mg/kgm/day for 2 months, followed by a taper of 8 mg/month for 6–8 months). The study had to be halted due to serious adverse events, including two treatment-related deaths; however, it did show clinical benefit, leading to TESTING 2.0. TESTING 2.0 used a lower dose of glucocorticoids (oral methylprednisolone 0.4 mg/kg daily) along with *Pneumocystis jirovecii* prophylaxis. The mean baseline eGFR was 61.5 mL/min/1.73m^2^ and mean proteinuria was 2.4 gm/day. Median time since kidney biopsy was 5 months (range 4–27 months). The primary composite outcome (i.e., 40% eGFR reduction, kidney failure, or death due to kidney disease) occurred less frequently in the methylprednisolone group than in the placebo group [74 (28.8%) vs. 106 (43.1%), respectively, HR 0.53 (95% CI 0.39 to 0.72), *p* < 0.001] over a mean follow-up of 4.2 years. A beneficial effect was observed in both the high- and reduced-dose groups when analyzed separately. Serious adverse events (hospitalization due to serious infection, gastrointestinal bleeding, clinically evident fractures, osteonecrosis, and new-onset diabetes mellitus) were significantly higher in the methylprednisolone group (37 vs. 8 total events, 10.9% vs. 2.8% subjects), including four fatalities (three in the high-dose group, one in the reduced-dose group). Among serious adverse events, 30 occurred in the high-dose group, seven in the reduced-dose group, and eight in the placebo group. Twelve patients in the high-dose group had severe infections requiring hospitalization compared with five patients in the reduced-dose group and three patients in the placebo group. All four cases of *Pneumocystis* pneumonia occurred in the high-dose steroid group. The reduced-dose group had no gastrointestinal bleeding, fractures, or osteonecrosis reported (29, 30).

The higher proteinuria in TESTING participants compared with STOP-IgA participants (2.4 vs. 1.7 gm/day), higher annual eGFR loss (4.97 vs. 2.68 mL/min/1.73 m^2^/year), and higher MEST-C scores (M1 lesions: 59.8% vs. 25.7%, E1 lesions: 25.2% vs. 17.1%) indicate a greater benefit of steroids in patients who are at high risk of disease progression.

It is important to note that in most clinical trials there is an interval time period between retrieving the kidney biopsy and clinical trial enrollment. Two specific elements related to this approach need to be considered. First, only a diagnosis of biopsy-proven IgAN was needed, without consideration of the amount of activity versus chronicity in the biopsy specimen. Second, the longer the time interval between biopsy and treatment initiation, the higher the likelihood of progression to chronic disease, which may not be as responsive to immunosuppressive therapy. Also, most of the trials require a RAAS-i/run-in phase of at least 3 months (consistent with KDIGO guidelines) prior to randomization, allowing time for possible progression to chronic histologic disease (e.g., in STOP-IgA, mean time from biopsy to trial enrollment was 5 months along with a 6-month run-in period). Allowing a longer interval period (between biopsy and treatment initiation) and mixing acute and chronic pathologic changes may bias trial results to the null, even if potentially beneficial for patients with recently diagnosed highly active IgAN.

There are a few ongoing trials that aim to minimize the interval between biopsy and treatment initiation along with stratifying patients into groups according to renal histology findings (active vs. chronic lesions). For example, the TIGER trial (NCT03188887), for which the inclusion criteria included renal biopsy within 45 days of inclusion visit, compared steroids plus RAAS-i or SGLT2 inhibitors with RAAS-i or SGLT2 inhibitors along with repeat biopsy at 12 and 24 months to compare the evolution of histologic lesions. The CLIgAN trial (NCT04662723) is composed of two RCTs; the first RCT will compare RAAS-i plus steroids with RAAS-i only in patients with active renal lesions and the second RCT will compare RAAS-i plus SGLT2 inhibitors with RAAS-i alone in patients with chronic renal lesions. Another trial is investigating the relationship between MEST-C classification and clinical remission rates after treatment (NCT05528991). The results of these trials will be highly anticipated and will help the physician in choosing the optimal candidate for steroid therapy.

The KDIGO 2021 guidelines state that patients with persistent proteinuria > 1gm/day despite optimized SC for 3–6 months should be offered clinical trial enrollment as the first step and a 6-month course of glucocorticoid therapy as the next step; however, with the caveat that the clinical benefit of glucocorticoids in IgAN is not established, and glucocorticoid therapy should be given with extreme caution or avoided entirely in patients with an eGFR of < 30 mL/min/1.73 m^2^, diabetes, obesity (BMI > 30 kg/m^2^), active peptic ulcer disease, latent infections (e.g., viral hepatitis, TB), secondary disease (e.g., cirrhosis), severe osteoporosis, and/or uncontrolled psychiatric illness (5). The KDIGO guidelines emphasize discussing the risk of steroid-related adverse effects with patients (serious infections leading to hospitalization, gastrointestinal bleeding, new-onset impaired glucose tolerance/diabetes, weight gain, osteonecrosis, and fractures), particularly those with an eGFR of < 50 mL/min/1.73 m^2^ (category 2B recommendation: 2: We suggest (rather than recommend), B: The true effect is likely to be close to the estimate of the effect, but there is a possibility that it is substantially different.), and encourage “incorporating prophylaxis against Pneumocystis pneumonia along with gastroprotection and bone protection when using glucocorticoids (prednisone equivalent of 0.5 mg/kg/day)”. It also states that “there is no data to support efficacy or reduced toxicity of alternate-day glucocorticoid regimens, or dose-reduced protocols”. It is highly imperative to note that the 2021 KDIGO guidelines were published before TESTING 2.0 was completed and thus do not take the reduced-dose protocol results into consideration. Therefore, low-dose steroids can be considered as one of the options prior to clinical trial enrollment. Future trials evaluating the efficacy and safety of combination of steroids and SGLT2 inhibitors would be of value, since SGLT2 inhibitors are becoming the standard of care and pose an increased risk of ketoacidosis and genitourinary infections.

### Mycophenolate mofetil

The use of mycophenolate mofetil (MMF) is currently recommended only in Chinese patients as a steroid-sparing agent, as per KDIGO guidelines. A randomized clinical trial conducted in China showed non-inferiority of MMF plus low-dose steroids compared with standard-dose steroids, along with significantly fewer side effects in the combination group (31). A Chinese trial comparing MMF with steroids showed greater reduction in proteinuria in the MMF group (32). However, two other RCTs conducted in Belgium and the USA showed no beneficial effects (33, 34). A longer trial in Hong Kong (40 Chinese patients, 6 years’ follow-up), which compared MMF for 6 months with SC, demonstrated a slower annual decline in eGFR, 1.12 mL/min/1.73 m^2^/year in the MMF group compared with 3.81 mL/min/1.73 m^2^/year in the SC group (*p* = 0.012) (35). A reduction in proteinuria in the MMF group was also observed, but only during the first 24 months.

The MAIN trial compared MMF (1.5 gm/day for the first 12 months followed by a 0.75–1.0 g maintenance dose) with SC (maximally tolerated losartan and low salt diet) in 170 Chinese adults with proteinuria > 0.75 gm/day and a eGFR between 30 and 60 mL/min/1.73 m^2^ after a 3-month run-in phase (36). Primary outcomes were a composite of doubling of creatinine, ESRD, death due to kidney or cardiovascular cause, and progression of CKD. The primary composite outcome was observed in six (7.1%) patients in the MMF group and 18 (21.2%) patients in the SC group [*p* = 0.008, HR 0.23 (95% CI 0.09 to 0.63)]. Progression of CKD occurred in seven (8.2%) patients in the MMF group and 23 (27.1%) patients in the SC group [*p* = 0.001, HR 0.23 (95% CI 0.10 to 0.57)]. The MMF group also had a higher reduction in proteinuria than the SC group (57.1% vs. 28.2%, *p* < 0.001). During the post-trial follow-up, mean annual eGFR loss was 7.1 mL/min/1.73 m^2^ in the SC group and 6.1 mL/min/1.73 m^2^ in patients who discontinued MMF after the trial compared with 4.1 mL/min/1.73 m^2^ in patients who continued MMF during the post-trial period. Serious adverse events occurred more frequently in the MMF group; however, they were not statistically different (*p* = 0.37). Infections, especially pneumonia, and elevated liver enzymes were more common in the MMF group, but also not statistically significant (*p* = 0.37 and *p* = 0.21, respectively). Gastrointestinal side effects were the only adverse effects that were statistically different between the two groups (*p* = 0.001).

Both the Hong Kong trial and MAIN trial showed that discontinuation of MMF compared with continuing maintenance MMF resulted in accelerated eGFR decline, underscoring the importance of continuation of therapy. The Hong Kong trial did not include patients with advanced glomerulosclerosis, tubular atrophy, or interstitial fibrosis; however, in the MAIN trial, 83.5% of patients had MEST-C score of S1 and 54.1% had a score of T2, showing benefit in patients with advanced histologic disease as well. Using low-dose MMF (1.5 gm/day for the first 12 months followed by a 0.75–1.0 g maintenance dose vs. 2 gm/day or 3 gm/day) resulted in lower infectious complications (16.5% vs. 54% and 79%, respectively) (37, 38). The role of MMF as an immunosuppressant in patients with contraindications to steroids or as a low-dose maintenance therapy needs to be explored further in non-Chinese patients.

### Targeted-release budesonide

Targeted-release formulation of budesonide (TRF-budesonide) is an oral formulation of budesonide that is designed to deploy and release at the terminal ileum, thus delivering the medication locally to Peyer’s patches (gut-associated lymphoid tissue), which is a primary site for IgA production. The local delivery system minimizes systemic exposure with a potential for reduced systemic side effects of steroids (39). The FDA approved the oral, delayed-release, targeted formulation of budesonide designed for IgAN in December 2021 because of its efficacy in reducing proteinuria (long-term effects on eGFR were still to be determined since Nefigard Part B was ongoing at that time).

The Phase II Nefigan trial evaluated budesonide 8 mg or 16 mg compared with placebo for 9 months. Use of budesonide for 9 months was associated with a 24.4% decrease from baseline in mean UPCR (change in UPCR vs. placebo 0.74; 95% CI 0.59 to 0.94; *p* = 0.0066). At 9 months, mean UPCR had decreased by 27% in patients who received 16 mgm/day (0.71; 95% CI 0.53 to 0.94; *p* = 0.0092) and 21.5% in patients who received 8 mgm/day (0.76; 95% CI 0.58 to 1.01; *p* = 0.0290), whereas patients who received placebo had an increase in mean UPCR of 2.7%. Incidence of adverse events was similar in all groups (88% in the TRF-budesonide 16 mgm/day group, 94% in the TRF-budesonide 8 mg/day, and 84% in placebo group), with nasopharyngitis being the most common adverse event (40). The promising results led to the Phase III Nefigard trial.

Similar to its predecessor, Part A of the Nefigard trial showed that patients receiving optimized RAAS-i along with TRF-budesonide (Nefecon 16 mg) for 9 months had a 48% reduction in UPCR compared with placebo (*p* < 0.0001) at 12 months. After 9 months of treatment, eGFR in the Nefecon group decreased from baseline by 0.17 mL/min/1.73 m^2^ compared with 4.04 mL/min/1.73 m^2^ in the placebo group. The difference was maintained at 12 months with a 3.37 mL/min/1.73 m^2^ per year (*p* = 0.01) improvement in eGFR (41). Unlike the reduction in proteinuria, the greatest benefit in eGFR was seen in the subgroup with baseline UPCR ≥ 1.5 g/g, and there was no benefit in patients with baseline UPCR < 1.5 g/g.

Results of Part B of the Nefigard trial were recently published with findings consistent with Part A. The primary end point, eGFR over 2 years, was on average 5.05 mL/min/1.73 m^2^ higher with Nefecon than with placebo (*p* < 0.0001). Mean change in eGFR over the 2-year period was -2.47 mL/min/1.73 m^2^ for Nefecon 16 mg compared with -7.52 mL/min/1.73 m^2^ for placebo (percentage change of –11% in Nefecon group vs. –21.5% in placebo group). The percentage change in UPCR at 24 months was –30.7% in the Nefecon group compared with –1.0% in the placebo group. Treatment benefit on eGFR was observed across baseline UPCR subgroups. The drug was well tolerated, with the most common adverse events being peripheral edema, hypertension, muscle spasms, and acne. Less than 10% of patients discontinued the drug due to adverse events. The study concluded that a prolonged positive effect on proteinuria and eGFR was observed, even after 15 months of drug discontinuation, indicating a disease-modifying capability.

### Other immunosuppressants

As per KDIGO guidelines, there is no documented evidence of efficacy for azathioprine, cyclophosphamide (unless rapidly progressive IgAN), calcineurin inhibitors, or rituximab (5). In the Stop-IgA trial, the arm with an eGFR of 30–59 mL/min/1.73 m^2^ did receive oral cyclophosphamide along with oral steroids for 3 months, but, as mentioned above, it did not have any effect on long-term outcomes (27).

## Fish oil

There is conflicting evidence about the use of fish oil in IgAN. As per KDIGO guidelines, patients who wish to take fish oil should be advised of the dose and formulation used in the published clinical trials that reported efficacy. A multi-center randomized clinical trial comparing fish oil (12 g daily) with a similar dose of olive oil in patients with IgAN who had persistent proteinuria showed that, after a mean follow-up of 6.4 years, 17 patients in the fish oil group and 29 in the placebo group reached the primary end point (50% reduction in serum creatinine; *p* = 0.002), and eight patients in the fish oil group and 19 in the placebo group developed ESRD (*p* = 0.009) (42, 43). It concluded that early and prolonged treatment with fish oil slows disease progression for high-risk patients with IgAN. Another RCT tested a 6-month course of polyunsaturated fatty acids (PUFA) (3 gm/day) in a group of 30 patients with biopsy-proven IgAN and proteinuria already treated with RAAS-i compared with continuing standard therapy. At the end of the 6-month trial, the reduction in proteinuria was 72.9% in the PUFA group and 11.3% in the RAAS group (*p* < 0.001). A reduction of ≥ 50% of baseline proteinuria was achieved in 80.0% of PUFA patients and in 20.0% of RAAS-i patients (*p* = 0.002). Low- and high-dose omega-3 fatty acids (eicosapentaenoic acid (EPA) 1.88 g and docosahexaenoic acid (DHA) 1.47 g vs. EPA 3.76 g and DHA 2.94 g) have been shown to be similar in slowing the rate of renal function loss (44). Doses should be 3.3 gm/day or more of prescription-strength omega-3 fatty acids, and not over-the-counter supplements.

## Tonsillectomy

As per KDIGO guidelines, tonsillectomy should not be performed as a treatment for IgAN in Caucasian patients because there is paucity of data in this population. The data that are available do not support the efficacy of tonsillectomy in Caucasian and Chinese patients. However, in Japan, it is routinely performed because multiple studies have reported improved kidney survival and partial or complete remission of hematuria and proteinuria following tonsillectomy alone or with pulsed glucocorticoids (45–47).

## IgA nephropathy and pregnancy planning

All women of childbearing potential should be offered preconception counseling when appropriate. Preconception counseling should include a discussion on cessation of RAAS inhibitors and blood pressure control with alternative antihypertensives preconception. In those women at high risk of progressive CKD despite maximal supportive care, a trial of immunosuppression to reduce proteinuria prior to conception may be preferable to emergent initiation of immunosuppression during pregnancy.

## Novel treatments and ongoing trials

There are several novel treatments that have been discovered and are currently being studied for IgAN. A search for ongoing clinical trials in IgAN was conducted *via* ClinicalTrials.gov until March 2023 and has been summarized in **Table 2**.

## Discussion

**Figure 1:**
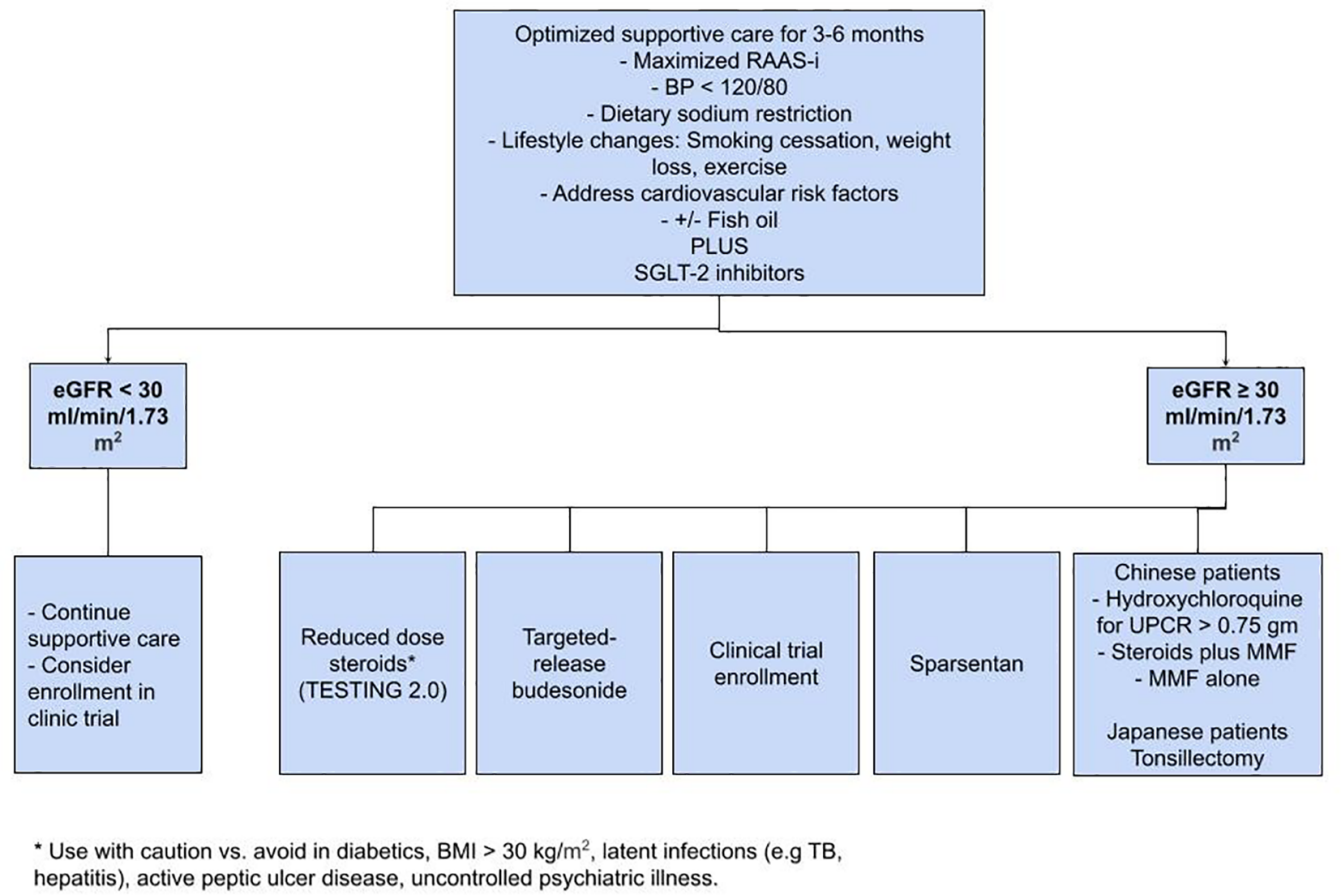
Management of patients with IgA nephropathy.

IgAN continues to be the most prevalent glomerular disease worldwide. As a first step, SC, including RAAS-i and SGLT2 inhibitors, plays a paramount role in slowing the progression of the disease. For patients at high risk of progression, low-dose steroids, targeted budesonide, and sparsentan appear promising; however, it is extremely important to discuss appropriate clinical trials and possible enrollment with the patient as well (**Figure 1**). The results of the trials mentioned in **Table 2** could significantly alter the future of IgAN’s treatment paradigm. Commencing early supportive care, evaluating the need for immunosuppression, and timely referral to clinical trials has the potential to change the course of disease in patients with IgAN.

**Table 2:** Clinical trials of novel medications in IgAN.

Trial (NCT number)	Intervention	Mechanism of intervention	Inclusion criteria	Exclusion criteria	Trial design; status	Primary end point (results)	Follow-up duration
Glucocorticoids							
NefIgArd (NCT03643965)	Nefecon 16 mg once daily by mouth for 9 months vs. matching placebo	A modified-release formulation of budesonide that targets the sites of mucosal B-cell induction	Proteinuria ≥ 1 gm/day Stabilized on RAAS-i at the maximum tolerated dose according to 2012 KDIGO guidelines eGFR 35–90 mL/min/1.73 m^2^ in using CKD-EPI equation	Acute or chronic infectious disease Unacceptable blood pressure control Liver cirrhosis	Randomized, double-blind, placebo-controlled Phase III Ongoing	Change in proteinuria in 9 months eGFR in 2 years	2 years
PL-56 in IgAN (NCT00767221)	Budesonide 8 mg once daily for 6 months	Corticosteroid	Albuminuria > 500 mg/day Serum creatinine < 200 μmol/L	Use of investigational drug within 30 days Using cytochrome P450 enzyme inhibitors	Open-label Phase II Completed	Albuminuria at 3 months (No publication posted)	9 months
Complement pathway inhibitors							
CCX168 in IgAN (NCT02384317)	Avacopan twice daily for 48 days	C5a receptor inhibitor	Proteinuria > 1 g/g Stabilized on RAAS-i eGFR > 60 mL/min/1.73 m^2^	Use of immunosuppressants within 24 weeks Henoch–Schönlein purpura with systemic manifestations within 2 years Proteinuria > 8 g/g	Open-label Phase II Completed	Incidence of adverse events at 169 days (No publication posted)	169 days
LNP023 in kidney disease caused by inflammation (NCT03373461)	Iptacopan twice daily (part 1: 10 mg, 50 mg, 200 mg; part 2: 10 mg, 50 mg, 100 mg, and 200 mg) vs. matching placebo	A factor B inhibitor of the alternative complement pathway	Proteinuria ≥ 1 gm/day at screening and ≥ 0.75 gm/day after the run-in period Stabilized on RAAS-i for at least 90 days eGFR ≥ 30 mL/min/1.73 m^2^	Use of immunosuppressants within 90 days or other investigational drugs within 30 days Plasma donation within 30 days History of porphyria metabolic disorder History of alcohol or drug abuse within 12 months	Randomized, double-blind, placebo-controlled Phase II Completed	Reduction of proteinuria at 90 days (No publication posted)	180 days
Narsoplimab in IgAN (NCT02682407)	Narsoplimab vs. matching placebo	Human monoclonal antibody against mannan-associated lectin-binding serine protease-2 (MASP-2)	Proteinuria > 1 gm/day Stabilized on RAAS-i for 6 months	Use of investigational drugs within 6 months	Open-label Phase II Ongoing	Treatment-related adverse events Change from baseline in urine and serum complement components	104 weeks
ALXN2050 in LN & IgAN (NCT05097989)	Vemircopan 120 mg vs. Vemircopan 180 mg vs. placebo in IgAN cohort	Factor D inhibitor	Proteinuria ≥ 1 gm/day Presence of hematuria Stabilized on RAAS-i for ≥ 3 months	Use of glucocorticoid within 6 months Blood pressure > 140/90 mmHg Rapidly progressive glomerulonephritis diagnosis within 3 months	Randomized, multi-center, double-blind, placebo-controlled Phase II Ongoing	Change of proteinuria at week 26	50 weeks
SANCTUARY in LN & IgAN (NCT04564339)	Ravulizumab based on body weight	Terminal complement pathway inhibitor	Proteinuria ≥ 1 gm/day Stabilized n RAAS-i for 3 months	Use of glucocorticoid within 6 months Previous use of complement inhibitor	Randomized, double-blind, placebo-controlled Phase II Ongoing	Change of proteinuria at 26 weeks	50 weeks
B-cell inhibitors							
BRIGHT-SC (NCT02062684)	Subcutaneous blisibimod 100 mg three times weekly for 8 weeks then 200 mg weekly for 16 weeks vs. matching placebo	A selective peptibody antagonist of B-cell activating factor	Proteinuria ≥ 1 gm/day but ≤ 6 gm/day at two consecutive time points Stabilized on RAAS-i for 8 weeks	Use of glucocorticoid within 3 months or immunosuppressants within 6 months Neutropenia	Randomized, double-blind, placebo-controlled Phase II/III Completed	Reduction in proteinuria at 42 weeks (No publication posted)	104 weeks
RC18 in IgAN (NCT04291781)	Subcutaneous telitacicept 160 mg once weekly for 24 doses vs. 240 mg once weekly for 24 doses vs. matching placebo	A fusion protein that neutralizes the B lymphocyte stimulator and a proliferation-inducting ligand	Proteinuria ≥ 1 gm/day Stabilized on RAAS-i for 4 weeks eGFR > 45 mL/min/1.73 m^2^	Use of glucocorticoid within 6 months or investigational drug within 4 weeks Cytopenia A cardiovascular event within 12 weeks	Randomized, double-blind, placebo-controlled Phase II Completed	Reduction of proteinuria at week 24 (No publication posted)	24 weeks
ORIGIN (NCT04716231)	Atacicept weekly subcutaneous injections vs. matching placebo	Recombinant fusion protein that inhibits B lymphocytes stimulators and a proliferation-inducing ligand	Proteinuria > 0.75 gm/day Stabilized on RAAS-i for 12 weeks	Rapidly progressive glomerulonephritis Nephrotic syndrome	Randomized, double-blind, placebo-controlled Phase IIb Ongoing	Reduction of proteinuria at 24 weeks	2 years
Telitacicept in IgAN (NCT05596708)	Telitacicept 240 mg weekly subcutaneous injection for 104 weeks vs. matching placebo	Recombinant fusion protein that inhibits B lymphocytes stimulators and a proliferation-inducing ligand	Proteinuria ≥ 0.75 gm/day Stabilized on RAAS-i for 12 weeks	Use of immunosuppressants within 3 months	Randomized, double-blind, placebo-controlled Phase III Ongoing	Complete clinical response The absolute value of eGFR Reduction of proteinuria	3 years
VISIONARY (NCT05248646)	Sibeprenlimab 400 mg subcutaneous every 4 weeks vs. matching placebo	Humanized monoclonal IgG2 antibody that inhibits a proliferation-inducing ligand	Proteinuria ≥ 1 gm/day Stabilized on RAAS-i for 3 months	Use of immunosuppressants within 16 weeks Nephrotic syndrome	Randomized, multi-center, double-blind, placebo-controlled Phase III Ongoing	Reduction of proteinuria at 9 months	24 months
BION-1301 in IgAN (NCT03945318)	BION-1301 vs. placebo, 2 parts	Humanized monoclonal antibody against a proliferation-inducing ligand	Proteinuria ≥ 0.5 gm/day Stabilized on RAAS-i for 3 months	Use of glucocorticoid within 3 months	Randomized, multi-center, double-blind, placebo- controlled Phase I/II Ongoing	Incidence of treatment-emergent adverse events and their severity	76 weeks
IGANZ (NCT05065970)	Felzartamab vs. matching placebo	Anti-CD38+ monoclonal antibody	Proteinuria ≥ 1 gm/day Stabilized on RAAS-i for ≥ 3 months	Diabetes mellitus type 1 Deranged liver enzymes Cytopenia	Randomized, multi-center, double-blind, placebo-controlled Phase IIa Ongoing	Change of proteinuria at 9 months	2 years
AT-1501 in IgAN (NCT05125068)	Tegoprubart 10 mg/kg vs. 5 mg/kg both every 3 weeks for 93 weeks, a total of 32 infusions	Anti-CD40L monoclonal antibody	Proteinuria ≥ 0.75 gm/day Stabilized on RAAS-i for at least 90 days	Diabetes mellitus Blood pressure > 140/90 mmHg	Non-randomized, multi-center, open-label Phase IIa Ongoing	Change of proteinuria at 24 weeks Safety	100 weeks
Rituximab in IgAN (NCT00498368)	Intravenous rituximab 1 g on days 1 and 15, and the course repeated after 6 months vs. matching placebo	A chimeric monoclonal antibody directed against the CD20 antigen of B cells	Proteinuria ≥ 1 gm/day while on stable RAAS-i for 2 months eGFR 30–90 mL/min/1.73 m^2^	Use of glucocorticoid for > 6 months or previous treatment with rituximab or natalizumab History of Crohn’s disease or celiac sprue	Randomized, multi-center, open-label Phase IV Completed	Reduction of proteinuria at 12 months [Results showed that rituximab did not significantly improve renal function or proteinuria assessed over a year (48),]	12 months
RITA in IgAN (NCT04525729)	Rituximab (1 g on day 1, day 31, and at 6 months) and RAAS-i vs RAAS-i	A chimeric monoclonal antibody directed against CD20 antigen of B cells	Stabilized on RAAS-i for 3 months	Use of glucocorticoid within 12 months or other immunosuppressants within 6 months	Randomized, multi-center, controlled	Change of proteinuria at 1 year	1 year
Protein degradation inhibitors							
Velcade in IgAN (NCT01103778)	Intravenous bortezomib 1.3 mg/m^2^ on days 1, 4, 8, and 11. A second cycle is to be given for non-responders 1 month later	A proteasome inhibitor	Proteinuria > 1 gm/day Stabilized on RAAS-i for at least 4 weeks	Use of investigational drug within 14 days Low platelet and neutrophil count Peripheral neuropathy	Open-label Phase IV Completed	Reduction of proteinuria at 12 months [Results showed that 38% had achieved the primary end point at 1-year follow-up (49)]	12 months
SIGN (NCT02112838)	Fostamatinib 150 mg twice daily vs. fostamatinib 100 mg twice daily vs. matching placebo	A relatively selective small molecule spleen tyrosine kinase inhibitor	Proteinuria > 1 gm/day at diagnosis of IgAN Stabilized on RAAS-i for at least 90 days eGFR > 30 mL/min/1.73 m^2^	Recent use of immunosuppressants or > 15 mg/day of prednisone	Randomized, multi-center, double-blind, placebo-controlled Phase II Completed	Reduction of proteinuria at week 24 (No publication posted)	24 weeks
ANG-3070 in chronic kidney disease (NCT04939116)	ANG-3070 200 mg once daily vs. 400 mg once daily vs. 300 mg twice daily vs. placebo for 12 weeks	Oral tyrosine kinase inhibitor	Proteinuria ≥ 1 gm/day Stabilized on RAAS-i eGFR ≥ 40 mL/min/1.73 m^2^	Deranged liver enzymes Type 1 diabetes Positive hepatitis B or C or HIV viral screening	Randomized, multi-center, double-blind, placebo-controlled Phase II Ongoing	Change of proteinuria at 12 weeks	12 weeks
Endothelin receptor antagonists							
PROTECT (NCT03762850)	Sparsentan 400 mg total *vs.* irbesartan 300 mg total for 110 weeks	Dual endothelin angiotensin receptors antagonist	Proteinuria ≥ 1 gm/day Stabilized on RAAS-i for 12 weeks Blood pressure ≤ 150/100 mmHg	Use of glucocorticoid within 3 months History of heart failure	Randomized, multi-center, double-blind, active-control Phase III Ongoing	Change of proteinuria at week 36	2 years
ALIGN (NCT04573478)	Atrasentan 0.75 mg orally daily for 132 weeks vs. matching placebo	Endothelin A antagonist	Proteinuria ≥ 1 gm/day Stabilized on RAAS-i for at least 12 weeks	Use of immunosuppressants for more than 2 weeks within 3 months	Randomized, multi-center, placebo-controlled Phase III Ongoing	Change in proteinuria at week 24	2.6 years

## Author contributions

SMN wrote the review article. FA created the clinical trials table. SN wrote the abstract. SN, RM, ZZ, and AA reviewed the article. All authors contributed to the article and approved the submitted version.
